# Comparison of amino acid digestibility of soybean meal, cottonseed meal, and low-gossypol cottonseed meal between broilers and laying hens

**DOI:** 10.5713/ab.22.0073

**Published:** 2022-09-02

**Authors:** Kai Qiu, Xiao-cui Wang, Jing Wang, Hao Wang, Guang-hai Qi, Hai-jun Zhang, Shu-geng Wu

**Affiliations:** 1 National Engineering Research Center of Biological Feed, Institute of Feed Research, Chinese Academy of Agricultural Sciences, Beijing 100081, China

**Keywords:** Amino Acid Digestibility, Broiler, Cottonseed Meal, Laying Hen, Soybean Meal

## Abstract

**Objective:**

This study aimed to determine and compare the apparent ileal digestibility (AID) and the standardized ileal digestibility (SID) of amino acids (AA) in soybean meal (SBM), cottonseed meal (CSM), and low-gossypol cottonseed meal (LCSM) fed to broiler chickens and laying hens.

**Methods:**

Three semi-purified diets containing the identical crude protein concentration at 20% were formulated to contain SBM, CSM, or LCSM as the sole source of N. A N-free diet was also formulated to estimate the basal ileal endogenous losses of AA for broilers and hens. A total of 300 male Ross 308 chicks at one-day-old and 144 Hy-Line Brown laying hens at 30-week-old with initial egg production rate of 88.3%±1.0% were randomly allocated into 1 of 4 dietary treatments, respectively.

**Results:**

CSM and LCSM showed more Arg and Cys+Met while less Lys, Ile, Leu, and Thr relative to SBM. Significant interactions existed between species and experimental diets for AID (except for Arg, Asp, Glu, Gly, and Pro) and SID (except for Arg, His, and Phe) of most AA. Most AA in diets showed higher AID (except for Lys) and SID (except for Lys, Met, and Ser) in broilers relative to laying hens. The AID and SID of all AA were significantly different between the three diets. In broilers, the AID and SID of most indispensable AA except for Arg in SBM and LCSM was higher than CSM. In laying hens, the AID and SID of most indispensable AA except for Arg, Met+Cys, and Phe in SBM was higher than CSM and LCSM.

**Conclusion:**

The accurate determination of AID and SID of AA in CSM and LCSM for broilers and layers benefits the application of CSM and LCSM in chicken diets. The cottonseed by-products CSM or LCSM showed the species-specific AA digestibility values for broilers and layers.

## INTRODUCTION

In livestock production, soybean meal (SBM), the most common protein ingredient, is widely used in animal diets [1]. However, with the rising costs and fluctuant availability of SBM over the years, alternative protein sources are increasingly explored and employed to reduce the SBM use in the feed industry [2–4]. To optimize the inclusion of these protein ingredients without compromising the efficient production and welfare of animals, it is important to formulate nutrition-balanced diets on the basis of digestible amino acids (AA) whose values are considered to be the best measure of the AA availability of protein feedstuffs [5]. In addition, diet formulations based on ileal digestible AA can effectively reduce total nitrogen content of feed and maximize efficiency of protein conversion to avoid nitrogen losses to the environment [6]. Apparent ileal digestibility (AID) and standardized ileal digestibility (SID) have been widely applied for diet formulation owing to their additivity [7]. However, there is a dearth of information about AID or SID of AA for the alternative protein sources, particularly for a specific target animal.

Cottonseeds are one of the major oilseeds around the world, and one of their by-products of processing after extracting oil and removing lint and hull, cottonseed meal (CSM) containing 22% to 56% crude protein (CP) and 7.4 to 11.99 MJ/kg metabolizable energy, is an attractive promising plant protein replacer of SBM, owing that CSM is more economical than SBM and can provide abundant protein to meet the requirements for animals [8–10]. Free gossypol (FG), a phenolic aldehyde, in CSM was recognized as an anti-nutritional factor to reduce growth performance and increase mortality in broiler chickens, and decrease egg quality of laying hens [11,12]. Gossypol damages the capabilities of digestion, reproduction, etc., in humans and monogastric animals, which limits CSM use in animal diets [13]. However, health and performance of animals were not hampered and even could be improved if the FG in CSM-based diets was removed or detoxified effectively [14,15]. Coupled with the progress in processing techniques, cottonseed processors has improved the method of oil extraction, which has resulted in low-gossypol CSM (LCSM) [16].

Chemical composition, energy, and AA digestibility in several kinds of CSM fed to growing pigs was already precisely evaluated [17]. However, efficient application of CSM and LCSM in the chicken diets is still limited by the scarce data of their AA digestibility including AID and SID. In the poultry industry, specialized breeds of chicken have been bred to maximize production efficiency, which resulted in current separate systems for egg and meat production. Differences in physiology and rearing systems between broilers and laying hens probably bring out their differences in the digestibility of nutrients in feed. The microbial composition was significantly more abundant in the cecum of laying hens than those of broilers [18]. Broilers had higher SID of AA in SBM for most of the nonessential AA relative to laying hens [19]. AA digestibility of energy feedstuffs have been demonstrated to be significantly different between broilers and laying hens [20]. Therefore, the present study aimed to determine the AID and SID of AA in SBM, CSM, and LCSM fed to broilers and laying hens, and to compare their AA digestibility between broilers and laying hens.

## MATERIALS AND METHODS

### Animal care

The study procedures including broilers and laying hens experiments were reviewed and approved (AEC-CAAS-20191207) by the Animal Care and Use Committee of the Feed Research Institute of the Chinese Academy of Agricultural Sciences.

### Experimental design and bird management

A 2×3 factorial arrangement with two breeds of chicken (laying hens vs broilers) and three sources of CP (SBM vs CSM vs LCSM) was designed in the present study. SBM and CSM originated from hulling and oil extraction processes of soybeans and cottonseeds, respectively. LCSM was produced from cottonseed and characterized by the low FG content originated from a specific one time low-temperature extraction and a subsequent fractional extraction process with two solvents for removing gossypol. The FG, CP, and AA concentration of SBM, CSM, and LCSM are listed in Table 1. Before experiment, birds were fed the basal diets (Table 2). The composition of experimental diets is shown in Table 3. Three semi-purified diets containing the identical concentration of CP at 20% as-fed basis were formulated to contain SBM, CSM, and LCSM as the sole source of N. To calculate SID of AA, an N-free diet was also formulated to estimate the basal ileal endogenous losses (BEL) of AA for broilers and hens [21], so four experimental diets in total were prepared in this study. All experimental diets provided vitamins and minerals for birds to meet or exceed the nutritional requirements (NRC, 1994). For basal ileal endogenous AA losses and AA digestibility calculations using the index method, 5 g/kg chromic oxide (Cr_2_O_3_) was added to each diet as an ingestible marker. The experimental diets fed to broilers and laying hens were from the same batch.

A total of 300 male Ross 308 chicks were obtained at one-day-old from a commercial hatchery and raised in the chicken facility of the Institute (Beijing, China). Birds were fed a standard corn-SBM-based starter diet that met all the nutrient requirements until day 20. Broilers management and supplied nutrients were strictly adhered to the recommendations and specifications for the Ross 308 breed (Aviagen, 2014). On day 21, based on initial body weight, broilers were randomly assigned to 1 of 4 dietary treatments with 6 replicate cages and 10 birds per cage. One hundred and forty-four 30-week-old Hy-Line Brown laying hens with an initial egg production rate of 88.3%±1.0% were randomly allocated into 1 of 4 dietary treatments with 6 replicate cages and 6 hens per cage. Laying hens housing and handling procedures during the experiment were in accordance with the recommendations of Hy-Line International Online Management Guide (Hy-Line International, 2011). All birds including broilers and hens had free access to water and diets. Broilers and laying hens were fed either a standard corn-SBM starter diet or a regular laying hen diet, respectively, which was adequate in all nutrients, prior to the feeding of the experimental diets.

### Collection of ileal digesta

At the end of the 5-d feeding period, all birds were euthanized by intra-cardial injection of 1.5 mL 40 mg/kg sodium pentobarbitone. The small intestine was immediately exposed, and the contents of the entire ileum were flushed into plastic containers by distilled water. The ileum was defined as the portion of the small intestine from Meckel’s diverticulum to about 5 mm proximal to the ileo-caecal-colonic junction. Digesta within a replicate were pooled, resulting in 6 samples for each treatment. The digesta samples were freeze-dried and ground with an electric grinder and filtered through a 3 mm screen to ensure a homogeneous mixture for analysis.

### Chemical analysis

The concentration of dry matter in protein ingredients, diets and ileal digesta samples were determined by placing duplicate samples in a drying oven at 105°C for 24 h according to the AOAC method (method 934.01; AOAC 2006). Nitrogen (N) content was determined using an N analyzer (model Kjeltec-8100; FOSS Analytical Co., Ltd. Copenhagen, Denmark), and the concentration of CP was calculated using the conversion factor of 6.25. Diets and ileal digesta for AA analysis were prepared by acid hydrolysis according to the AOAC international method (2000; 982.30 E [A.B.C]). Briefly, approximately 100 mg of each sample was hydrolyzed in 4 mL of 6 M HCl (or BaOH for the analysis of Trp) for 24 h at 110°C under N atmosphere, followed by neutralization with 4 mL of 25% (wt/vol) NaOH, and then cooled to about 25°C. Performic acid oxidation at 0°C was carried out before acid hydrolysis to analyze the content of Met and Cys (sulfur-containing AA). The concentration of AA in the hydrolyzed samples was determined using an AA analyzer (model Biochrom-30; Biochrom Ltd, Cambridge, UK).

Chromium (Cr) concentration in the diets and ileal digesta samples were determined following nitric and perchloric acid wet-ash digestion (method 935.13; AOAC International, 2000). Cr in the digestion products was quantified by spectrophotometry (method 946.06; AOAO International, 20000) and absorbance read using a Dynex plate reader (Dynex Technologies Inc., Chantilly, VA, USA). The concentrations of FG in SBM, CSM, and LCSM were detected using spectrophotometer according to national standards of China (GB13086-1991). Except for AA, all other analyses were done in duplicate.

### Calculations

The AID of AA was calculated by the following formula using the Cr marker ratio in the diet and ileal digesta [22].


$$\text{AID of AA}=\frac{{\left(\text{AA}/\text{Cr}\right)}_{\text{d}}-{\left(\text{AA}/\text{Cr}\right)}_{\text{i}}}{{\left(\text{AA}/\text{Cr}\right)}_{\text{d}}}$$

Where, (AA/Cr)_d_ represent the ratio of AA and chromium in diet, and (AA/Cr)_i_ represent the ratio of AA and chromium in ileal digesta.

Basal ileal endogenous flow values (g/kg DMI) determined by feeding the N-free diet. The BEL of AA at the ileum was calculated as grams lost per kilogram of DM intake (DMI) [23].


$$\text{BEL of AA }(\text{g}/\text{kg DMI})=\frac{\text{AA in ileal digesta }(\text{g}/\text{kg})\times {\text{Cr}}_{\text{d}}(\text{g}/\text{kg})}{{\text{Cr}}_{\text{i}}\;(\text{g}/\text{kg})}$$

Where Cr_d_ = chromium diet and Cr_i_ = chromium in ileal digesta.

AID data of AA were then converted to the values of SID, using endogenous N and AA values determined from birds fed the N-free diet [24].


$$\text{SID}=\text{AID}+\frac{\left(\text{Basal EAA }(\text{g}/\text{kg DMI})\right)}{\text{Ingredient AA }(\text{g}/\text{kg DM})}$$

Where Basal EAA = basal endogenous AA flow, and ingredient AA = concentration of the AA in the ingredient.

### Statistical analysis

Data about AID and SID of AA were analyzed by a 2×3 factorial using the general linear model procedure of SAS for a randomized complete block design (SAS 9.1, SAS Inst. Inc., Cary, NC, USA). The model included the fixed effect of experimental diets, chicken species, and their associated two-way interaction. The AID or SID of AA in a given chicken breeds were subjected to one-way analysis of variance (ANOVA) with diets as the main variable to determine the differences between SBM, CSM, and LCSM. The BEL of AA for broilers and laying hens were analyzed by a Student’s t-test procedure. Differences were considered significant at p≤0.05. Data were presented as mean±standard error of mean.

## RESULTS

### Chemical analysis

The FG concentration, CP level and total AA content of SBM, CSM, and LCSM are presented in Table 1. CSM and LCSM were analyzed to contain 700.83 and 106.67 mg/kg FG respectively. The AA composition of SBM with 48.58% CP was similar to the NRC (2012) values of dehulled and solvent extracted SBM. LCSM showed higher CP (53.11%) and total AA content than CSM containing 40.91% CP. Three protein ingredients show significant difference in AA composition, such as CSM and LCSM containing more Arg and Cys+Met while less Lys, Ile, Leu, and Thr as compared with SBM. As shown in Table 4, the analyzed CP content in the experimental diets was close to the designed value of 20%. The analyzed AA concentrations in experimental diets were basically consistent with the calculated values based on diet formula and AA content of ingredients.

### Apparent ileal digestibility of amino acids

Tested diets were formulated using protein ingredients (SBM, CSM, and LCSM) to be the only source of dietary CP. As shown in Table 5, most AA in three experimental diets showed higher (p<0.05) AID in broilers relative to laying hens except for Lys (p = 0.526). The AID of all AA was significantly different between the three diets (p<0.05). Significant interactions (p<0.05) between species and experimental diets existed for AID of most AA except for Arg, Asp, Glu, Gly, and Pro, which was described in detail as follows according to the results of one-way ANOVA within species (Supplementary Table S1). In broilers, the AID of Arg was higher in LCSM than SBM, and the AID of Met+Cys was higher in SBM and LCSM than CSM. However, in laying hens, the AID of Arg and Met+Cys were not different between diets. The AID of His, Met, Met+ Cys, Phe, Trp, Thr, and Val in broilers fed SBM or LCSM was higher (p<0.05) relative to CSM. The AID of Ile, Leu, and Lys in broilers was in turn significantly (p<0.05) decreased from SBM, LCSM, to CSM. The AID of His, Ile, Leu, Lys, Met, Trp, Thr, and Val in laying hens fed CSM or LCSM was lower (p<0.05) as compared with those fed SBM. In laying hens, the AID of Phe in CSM was less (p<0.05) than that in SBM, while that in LCSM was not different from both LCSM and SBM. In addition, the AID of indispensable AA (IAA), dispensable AA (DAA), and total AA (TAA) was significantly different (p<0.05) between both diets and species. The AID of IAA and TAA shows significant (p<0.05) interaction between diets and species.

### Basal ileal endogenous losses of amino acids

The determination of endogenous AA loss enables us to establish the AA SID values of feed ingredients by correcting the basic endogenous AA loss. The basal ideal endogenous AA flow of broilers and laying hens was detected using an N-free diet (Table 6). Both IAA and DAA showed more endogenous loss in laying hens as compared with broilers, including Lys, Met, Met+Cys, Ile, Thr, Trp, Val, Arg, His, Leu, Phe, Ala, Asp, Cys, Glu, Gly, Pro, and Ser.

### Standardized ileal digestibility of amino acids

As shown in Table 7, the SID of both IAA and DAA showed significantly (p<0.05) difference between experimental diets. There were significant (p<0.05) interactions for SID of most IAA except for Arg, His, and Phe, which was described in detail as follows according to the results of one-way ANOVA within species (Supplementary Table S2). Most AA in three experimental diets showed higher (p<0.05) SID in broilers relative to laying hens except for Lys, Met, and Ser. In broilers, the SID of Arg and Met+Cys for LCSM was higher (p<0.05) than CSM and similar with SBM. However, in laying hens, the SID of Arg and Met+Cys were not different between diets. The SID of His, Leu, Met, Phe, Trp, Thr, and Val in broilers fed SBM or LCSM was higher (p<0.05) than those fed CSM. The SID of Ile and Lys in broilers was in turn significantly (p<0.05) decreased from SBM, LCSM, to CSM. The SID of His, Ile, Leu, Lys, Met, Trp, Thr, and Val in laying hens fed CSM or LCSM was lower (p<0.05) than those fed SBM. In laying hens, the SID of Phe in CSM was less (p<0.05) than in SBM, while that in LCSM was not different from both LCSM and SBM. In addition, the SID of IAA, DAA, and TAA was significantly different (p<0.05) between both diets and species. The SID of IAA and TAA shows significant (p<0.05) interaction between diets and species.

## DISCUSSION

In animal feed, replacement of SBM with CSM or LCSM is an effective approach to reduce the feed cost and increase economic returns. In recent years, studies in our group have demonstrated that no more than half replacement of SBM with the by-products of cottonseed including CSM and cottonseed protein (about 300 mg/kg FG, lower than ordinary CSM) in chicken diets is feasible without negative effects on performance and egg quality [25,26]. However, the AID and SID of AA in CSM or LCSM for broilers and laying hens remains unevaluated, which obviously limits their tremendous and optimum utilization in chicken feed. Owing to nearly identical structure and function of the digestive tract between broilers and laying hens, the SID AA values of many feedstuffs presently used for laying hens were obtained from tests of cock of the breed of laying hens or directly referred to the data from broilers. In fact, the AA digestibility of broilers and laying hens were different because of their distinct age stage and purpose of production. Therefore, it is necessary to compare the AID and SID of AA in cotton meal between these two chicken breeds for precision feeding.

The CP level and AA composition of CSM in this study were consistent with the data of CSM in NRC (2012). The physicochemical and functional properties of proteins in CSM are influenced by its processing methods such as hot-pressed solvent extraction, cold-pressed solvent extraction and sub-critical fluid extraction [27–29]. Owing to the improved method of oil extraction and cottonseed processors, the CP level of LCSM in this study was up to 53.11%, significantly increased relative to CSM, and even exceeded SBM, which is close to the mean CP value of several solvent extracted CSMs in recent reports [17,30]. The FG concentrations of CSM were detected as 700.83 mg/kg which is within the scope of previously reported values (200 to 5,300 mg/kg) [9]. The FG level in LCSM was 106.67 mg/kg, far less than that of CSM. In addition, the AA composition characteristics indicating that CSM and LCSM contained more Arg and Cys+ Met but less Lys, Ile, Leu, and Thr in comparison with SBM should be taken into consideration once CSM is used to substitute SBM in diets. In the present study, the average SID of AA in CSM and LCSM for broilers was similar with those in CSM for poultry recommended by Chinese feed Composition and Nutritional Value Table (2017) and EVONIK (2016), while some of them are slightly higher than those for laying hens including Arg, His, Thr, Trp, and Val. Compared with the data of extracted-dehulled CSM for poultry in CVB Feed Table (2019) and INRAE-CIRAD-AFZ Feed Tables (https://www.feedtables.com), the average SID of His, Ile, Lys, and Val in CSM and LCSM for both broilers and laying hens obtained in the current study is a bit higher, while the other AA are similar, besides that, SID of Arg and Phe for broilers and SID of Leu for laying hens are shown slightly higher. Therefore, it indicated that the SID of AA in CSM for poultry is changed as the processing techniques of CSM progressed and shows obvious differences between broilers and laying hens, which also necessitate the current study.

Chickens can utilize a considerable amount of energy from canola meal, CSM, bakery meal, and peanut flour meal [31]. Taking AA digestibility into consideration, CSM produced from expander solvent could be fed to broilers at up to 21% in the complete ration [32]. In the current study, the AID and SID of AA in CSM was significantly lower than SBM for broilers, while LCSM had almost same values of AID and SID AA as SBM except for Lys and Ile. It indicated that LCSM probably has a wider space for use in broiler diets than CSM. The average SID of AA in broilers fed CSM in this study is almost similar with the value (71.7%) in a previous report [33]. The AID and SID of AA except for Arg in CSM in the present study were significantly lower than those in LCSM or SBM. It appears to be consistent with a study about growing pigs with lower AID and SID for Lys, Ile, Leu, Met, Thr, and Val in CSM as compared with the other protein sources [34]. Therefore, this observation indicated that FG would probably be the main factor that reduced AA digestibility of CSM in broilers. However, more investigations are needed to support this judgment.

In laying hens, dietary CSM decreased egg white protein synthesis in the magnum [12] and reduced egg production and feed efficiency [35], whereas, dietary LCSM supplementation of about 100 g/kg was recommended without adverse effects [25,36]. In this study, the AID and SID of most AA in laying hens fed CSM or LCSM was significantly lower than those fed SBM. The decreased FG concentration in CSM does not improve its AA digestibility in laying hens. It indicated that FG content in CSM was not the limiting factor affecting AA digestibility in laying hens, while the differences in the composition of AA between CSM, LCSM, and SBM may be the reason. After an expanded process, FG content in CSM decreased from 1.24 to 0.40 g/kg, and the LCSM can be used up to 10% in the total diet of laying hens without adversely impacting the performance [37]. Diets with CSM as the only protein source containing equivalent calculated nutrient content to the SBM diet showed no effects on egg production of Hy-Line Brown laying hens [38]. Therefore, it suggests that dietary nutrition especially AA balance should be firstly taken into consideration when CSM is used in the diets of laying hens, apart from its FG content.

The AID (except for Lys) and SID (except for Lys, Met, and Ser) of most AA in protein ingredients in this study was significantly lower in laying hens than broilers. It is consistent with the report that differences existed in the digestive capabilities of broilers and laying hens for protein ingredients, such as meat and bone meal and SBM [19], while contrary to a previous study that the AID of AA in SBM was higher for layers compared with those for broilers [39,40], which might result from different SBM quality and experimental animal breeds and age. Consistent with the results of Huang et al [39] and Adedokun et al [40], our results showed that the digestibility of most AA in CSM was similar between broilers and layers.

The significant interactions between species and experimental diets for AID and SID of most AA were observed in the current study, which might be owing to the differences existing in the digestive capabilities of laying hens and broilers [19,41] and the different relative tolerance to the toxicity of gossypol between laying and broiler breeder hens [42]. Health condition of laying hens is significantly worse than the case of meat-type poultry such as broilers and turkeys, especially in the impact on the liver due to the likely effect of nutrition [43]. The laying hen is more sensitive to gossypol ingestion than broilers, especially decreasing egg quality by brown yolk discoloration and then causing potential edible safety risk [44]. FG was demonstrated to directly affect follicular maturation and consequently female fertility [45]. In this study, reduction of the concentration of FG in the CSM could obviously increase the AID and SID of most AA in broilers, while this improvement was not found in laying hens. These results necessitate the investigations for the species-specific nutrient digestibility values of cottonseed by-products among different poultry species, which is indispensable for the explanation to the diverse performance response of different chickens exposed to varied levels of FG.

## CONCLUSION

This study accurately determined the AID and SID of AA in CSM and LCSM for broilers and laying hens and compared them with the regular protein source SBM. Replacement of SBM with CSM or LCSM in dietary formulations should take their distinct AA profiles into consideration. FG may be the main factor to reduce AA digestibility of CSM in broilers, whereas in laying hens, dietary AA balance should be firstly considered besides of FG content when CSM is used in the diets. The results of this study provided strong data supporting for the optimized application of CSM and LCSM in diets of broilers and laying hens.

## Supplementary Information





## Figures and Tables

**Table 1:** Analyzed nutrition contents in protein ingredients

Item	Protein ingredients		

	SBM	CSM	LCSM
Crude protein (%)	48.58	40.91	53.11
Dry matter (%)	89.89	89.58	92.22
Free gossypol (mg/kg)	-	700.83	106.67
Indispensable AA (%)			
Lys	2.922	1.735	2.098
Met	0.606	0.582	0.790
Cys+Met	1.391	1.324	1.783
Trp	0.628	0.481	0.651
Thr	1.878	1.326	1.644
Arg	3.476	4.754	6.154
His	1.298	1.177	1.479
Ile	2.200	1.304	1.552
Leu	3.569	2.323	2.828
Phe	2.561	2.304	2.972
Val	2.317	1.854	2.222
Dispensable AA (%)			
Ala	2.061	1.622	1.948
Asp	5.332	3.862	4.691
Cys	0.785	0.742	0.993
Glu	8.285	8.076	10.200
Gly	2.012	1.680	2.104
Pro	2.421	1.821	2.098
Ser	2.387	1.806	2.226

SBM, soybean meal; CSM, cottonseed meal; LCSM, low-gossypol cottonseed meal; AA, amino acids.

**Table 2:** Composition and nutrient levels of the basal diets, as-fed basis

Item	Broilers diet (1 to 20 d)	Laying hens diet
Ingredients (%)		
Corn	56.23	66.07
Soybean meal (47%)	34.37	22.83
Wheat-middlings	2.99	-
Soybean oil	2.27	-
Dicalcium phosphate	1.82	1.58
Limestone	1.31	8.80
Sodium chloride	0.30	0.30
DL-Methionine (98%)	0.24	0.17
L-Lysine-HCl (78%)	0.09	-
L-Threonine (98%)	0.06	-
Premix^1)^	0.22	0.13
Choline chloride (50%)	0.10	0.12
Total	100.00	100.00
Calculated nutrient levels		
Metabolizable energy (MJ/kg)	12.35	11.31
Crude protein (%)	21.50	16.50
Calcium (%)	1.00	3.50
Available phosphorus (%)	0.45	0.38
Lysine (%)	1.21	0.75
Methionine (%)	0.55	0.41
Methionine+cysteine (%)	0.88	0.65
Threonine (%)	0.86	0.55
Analyzed nutrient levels		
Crude protein (%)	21.43	16.67
Calcium (%)	1.09	3.62
Total phosphorus (%)	0.67	0.58

1) The premix used for broilers supplied the following per kg of complete feed: vitamin A, 12,500 IU; vitamin D_3_, 2,500 IU; vitamin K_3_, 2.65 mg; vitamin B_1_, 2 mg; vitamin B_2_, 6 mg; vitamin B_12_, 0.025 mg; vitamin E, 30 IU; Cu, 8 mg; Zn, 75 mg; Fe, 80 mg; Mn, 100 mg; Se, 0.15 mg; I, 0.35 mg; biotin, 0.0325 mg; folic acid, 1.25 mg; pantothenic acid, 12 mg; niacin, 50 mg. The premix used for laying hens supplied the following per kg of complete feed: vitamin A, 12,500 IU; vitamin D_3_, 4,125 IU; vitamin E, 15 IU; vitamin K, 2.0 mg; thiamine, 1 mg; riboflavin, 8.5 mg; calcium pantothenate, 50 mg; nicotinic acid, 32.5 mg; pyridoxine, 8 mg; vitamin B_12_, 5 mg; biotin, 2 mg; Fe, 60 mg; Mn, 65 mg; Se, 0.30 mg; I, 1.00 mg; Cu, 8 mg; Zn, 66 mg.

**Table 3:** Ingredient composition of experimental diets, as-fed basis

Item	Diets			

	SBM	CSM	LCSM	NFD
Crude protein (%)	20.01	20.00	20.02	0
Metabolizable energy (MJ/kg)	13.34	11.94	13.00	14.36
Ingredients (%)				
Corn starch	25.35	21.50	27.10	19.90
Dextrose	25.35	21.50	27.10	65.43
SBM	41.2	0	0	0
CSM	0	48.9	0	0
LCSM	0	0	37.7	0
Soybean oil	4.0	4.0	4.0	4.0
Monocalcium phosphate	1.9	1.9	1.9	1.9
Limestone	1.0	1.0	1.0	1.3
Chromic oxide^1)^	0.5	0.5	0.5	0.5
NaHCO_3_	0.2	0.2	0.2	1.2
Sodium chloride	0.2	0.2	0.2	0
Vitamin-mineral premix^2)^	0.22	0.22	0.22	0.22
Choline chloride	0.08	0.08	0.08	0.08
KCl	0	0	0	4
MgO	0	0	0	0.7
Cellulose^3)^	0	0	0	50
Total	100	100	100	100

SBM, soybean meal; CSM, cottonseed meal; LCSM, low-gossypol cottonseed meal; NFD, N-free diet.

1) Prepared by mixing 1 g of chromic oxide with 4 g of corn starch.

2) Provided per kilogram of diet: iron, 71.6 mg; copper, 11.0 mg; manganese, 178.7 mg; zinc, 178.7 mg; iodine, 3.0 mg; selenium, 0.4 mg; vitamin A (retinyl acetate), 18,904.3 IU; vitamin D_3_ (cholecalciferol), 9,480.0 IU; vitamin E (dl-α-tocopheryl acetate), 63.0 IU; vitamin K activity, 6.4 mg; thiamine, 3.2 mg; riboflavin, 9.4 mg; pantothenic acid, 34.7 mg; niacin, 126.0 mg; pyridoxine, 4.7 mg; folic acid, 1.6 mg; biotin, 0.5 mg; vitamin B_12_, 35.4 μg.

3) Purified cellulose, Tianjin Gugangfu Fine Chemical Research Institute, China.

**Table 4:** Analyzed nutrient content of experimental diets, as-fed basis

Item (%)	Diets			

	SBM	CSM	LCSM	NFD
CP (N×6.25)	21.43	23.47	20.37	0.00
Indispensable AA				
Lys	1.31	0.87	0.77	0.00
Met	0.27	0.29	0.26	0.00
Cys+Met	0.55	0.63	0.57	0.00
Trp	0.28	0.29	0.25	0.00
Thr	0.84	0.67	0.61	0.00
Arg	1.55	2.67	2.30	0.00
His	0.58	0.63	0.54	0.00
Ile	1.01	0.68	0.61	0.00
Leu	1.63	1.23	1.1	0.00
Phe	1.10	1.2	1.06	0.00
Val	1.04	0.96	0.86	-
Dispensible AA				
Ala	0.95	0.84	0.73	0.00
Asp	2.42	2.01	1.80	0.00
Cys	0.28	0.34	0.31	0.00
Glu	3.87	4.43	3.91	0.00
Gly	0.93	0.89	0.80	0.00
Pro	1.14	0.81	0.80	0.00
Ser	1.07	0.93	0.83	0.00

SBM, soybean meal; CSM, cottonseed meal; LCSM, low-gossypol cottonseed meal; NFD, N-free diet; CP, crude protein; AA, amino acids.

**Table 5:** Comparison of apparent ileal digestibility of AA between broilers and laying hens fed different ingredient samples

Item (%)	Diets			Species		SEM	p-value		
		
	SBM	CSM	LCSM	Broiler	Laying hen		Diets	Species	D×S
Indispensable AA									
Arg	87.11	85.84	88.25	88.25	85.88	0.78	0.015	0.001	0.220
His	82.54	75.01	77.99	81.01	76.01	1.07	<0.001	<0.001	0.032
Ile	81.13	64.72	70.66	74.46	69.88	1.40	<0.001	<0.001	<0.001
Leu	81.45	68.62	73.96	76.98	72.37	1.29	<0.001	<0.001	<0.001
Lys	83.11	60.75	66.25	70.45	69.62	1.59	<0.001	0.526	<0.001
Met	80.08	65.97	72.73	74.55	71.29	1.69	<0.001	0.025	<0.001
Met+Cys	71.95	66.20	72.14	73.20	66.99	1.53	0.001	<0.001	<0.001
Phe	82.88	78.15	81.75	83.30	78.55	0.99	<0.001	<0.001	0.041
Trp	79.55	69.82	73.85	76.28	72.52	1.30	<0.001	0.001	<0.001
Thr	73.03	57.48	64.08	68.37	61.35	1.79	<0.001	<0.001	<0.001
Val	78.35	68.18	72.62	76.03	70.06	1.37	<0.001	<0.001	0.003
Dispensable AA									
Ala	79.29	65.07	72.15	74.42	69.92	1.39	<0.001	<0.001	0.045
Asp	80.75	71.13	77.80	78.70	74.41	1.07	<0.001	<0.001	0.297
Cys	64.10	64.84	73.20	71.75	63.00	1.53	<0.001	<0.001	<0.001
Glu	85.96	81.65	86.08	86.25	82.87	0.79	<0.001	<0.001	0.887
Gly	76.16	64.56	72.10	73.34	68.53	1.33	<0.001	<0.001	0.065
Pro	80.83	66.69	76.02	77.76	71.26	1.25	<0.001	<0.001	0.106
Ser	78.01	66.38	74.06	75.20	70.42	1.45	<0.001	<0.001	0.018
IAA	81.10	72.78	76.96	79.02	74.87	1.20	<0.001	<0.001	0.001
DAA	81.40	73.62	79.73	80.43	76.07	1.05	<0.001	<0.001	0.208
Total AA	81.25	73.20	78.36	79.73	75.48	1.12	<0.001	<0.001	0.016

AA, amino acids; SBM, soybean meal; CSM, cottonseed meal; LCSM, low-gossypol cottonseed meal; SEM, standard error of mean; IAA, indispensable AA; DAA, dispensable AA.

**Table 6:** Basal ileal endogenous AA flow of 21-day-old broilers and 35 week-old laying hens fed an N-free diet (mg/kg DMI)

Item	Broiler	Laying hen	SEM	p-value
Indispensable AA				
Arg	224.00	367.52	17.48	<0.001
His	96.00	194.42	10.19	<0.001
Ile	195.49	339.28	16.43	<0.001
Leu	282.56	509.61	29.83	<0.001
Lys	224.00	363.02	18.79	<0.001
Met	74.60	113.01	5.64	<0.001
Met+Cys	202.80	347.33	19.89	<0.001
Phe	167.43	292.27	16.14	<0.001
Trp	69.36	131.53	5.88	<0.001
Thr	339.90	610.42	25.09	<0.001
Val	274.08	460.39	22.13	<0.001
Dispensable AA				
Ala	233.00	381.61	19.98	<0.001
Asp	448.34	763.74	37.87	<0.001
Cys	128.21	235.84	16.46	<0.001
Glu	565.65	894.41	46.70	<0.001
Gly	249.11	434.27	26.16	<0.001
Pro	250.96	454.63	35.54	0.002
Ser	249.11	434.27	26.16	<0.001
IAA	237.48	407.09	20.79	<0.001
DAA	195.47	338.98	15.61	<0.001
Total AA	303.48	514.11	29.20	<0.001

AA, amino acids; DMI, dry matter intake; SEM, standard error of mean; IAA, indispensable AA; DAA, dispensable AA.

**Table 7:** Comparison of standardized ileal digestibility of AA between broilers and laying hens fed different ingredient samples

Item (%)	Diets			Species		SEM	p-value		
		
	SBM	CSM	LCSM	Broiler	Laying hen		Diets	Species	D×S
Indispensable AA									
Arg	89.02	86.95	89.54	89.34	87.66	0.80	0.007	0.015	0.219
His	85.04	77.31	80.69	82.66	79.36	1.11	<0.001	0.001	0.052
Ile	83.78	68.65	75.04	77.13	74.52	1.44	<0.001	0.034	<0.001
Leu	83.88	71.84	77.56	79.18	76.34	1.33	<0.001	0.014	<0.001
Lys	85.35	64.12	70.06	72.84	73.50	1.62	<0.001	0.622	<0.001
Met	83.56	69.20	76.33	77.28	75.44	1.70	<0.001	0.191	<0.001
Met+Cys	76.95	70.56	76.97	76.69	72.96	1.64	<0.001	0.009	<0.001
Phe	84.97	80.06	83.92	84.80	81.16	1.03	<0.001	<0.001	0.061
Trp	84.24	74.36	79.11	81.11	77.36	1.36	<0.001	0.002	<0.001
Thr	78.69	64.58	71.86	73.27	70.15	1.80	<0.001	0.043	<0.001
Val	81.88	72.00	76.89	78.92	74.92	1.42	<0.001	0.002	0.005
Dispensable AA									
Ala	82.53	68.72	76.36	77.22	74.51	1.44	<0.001	0.028	0.063
Asp	83.26	74.14	81.16	80.89	78.14	1.12	<0.001	0.005	0.368
Cys	68.61	70.20	79.07	74.58	70.66	1.75	<0.001	0.001	<0.001
Glu	87.84	83.29	87.94	87.64	85.07	0.82	<0.001	0.001	0.900
Gly	79.83	68.41	76.37	76.21	73.52	1.40	<0.001	0.025	0.093
Pro	83.92	71.04	80.43	80.57	76.36	1.35	<0.001	0.001	0.154
Ser	82.17	71.16	79.42	78.58	76.58	1.51	<0.001	0.117	0.030
IAA	84.02	75.72	80.29	81.30	78.71	1.24	<0.001	0.016	0.002
DAA	84.19	76.51	82.96	82.62	79.81	1.11	<0.001	0.004	0.269
Total AA	84.11	76.11	81.64	81.97	79.27	1.17	<0.001	0.008	0.025

AA, amino acids; SBM, soybean meal; CSM, cottonseed meal; LCSM, low-gossypol cottonseed meal; SEM, standard error of mean; IAA, indispensable AA; DAA, dispensable AA.
