# Supplementary material for: Comparison of amino acid digestibility of soybean meal, cottonseed meal, and low-gossypol cottonseed meal between broilers and laying hens
Source: Anim Biosci. 2022 Sep 2;36(4):619–28. doi: 10.5713/ab.22.0073 (PMC9996273; doi:10.5713/ab.22.0073)
Supplement: Supplementary file 1 [file ab-22-0073-Supplementary-Table-1.pdf]

Table S1. The original data of apparent ileal digestibility of AA in broilers and laying hens fed different ingredient samples.

Item, %	Broiler			Laying hen		
	SBM	CSM	LCSM	SBM	CSM	LCSM
Indispensable AA						
Arg	88.04±0.91	86.49±0.89[ab]	90.22±0.41[a]	86.17±1.14	85.19±0.54	86.28±0.48
His	84.78±1.19[a]	76.17±1.50[b]	82.09±0.61[a]	80.30±1.29[x]	73.84±0.89[y]	73.89±0.62[y]
Ile	83.49±1.14[a]	63.36±2.34[c]	76.52±0.65[b]	78.77±1.60[x]	66.08±1.10[y]	64.79±0.91[y]
Leu	83.86±1.15[a]	67.65±2.11[c]	79.42±0.59[b]	79.04±1.52[x]	69.59±0.95[y]	68.49±0.76[y]
Lys	84.11±1.45[a]	56.61±2.85[c]	70.62±0.67[b]	82.10±1.52[x]	64.88±1.05[y]	61.87±1.05[y]
Met	82.94±1.69[a]	62.59±2.54[b]	78.13±0.77[a]	77.22±2.21[x]	69.34±1.20[y]	67.32±0.99[y]
Met+Cys	76.93±1.69[a]	63.64±2.17[b]	79.02±0.79[a]	66.96±2.02	68.75±1.15	65.26±0.70
Phe	85.17±0.95[a]	79.26±1.37[b]	85.48±0.47[a]	80.59±1.39[x]	77.04±0.74[y]	78.01±0.62[xy]
Trp	81.89±1.44[a]	67.91±1.93[b]	79.05±0.70[a]	77.20±1.54[x]	71.73±0.97[y]	68.64±0.75[y]
Thr	76.64±1.86[a]	56.10±3.05[b]	72.37±0.77[a]	69.42±1.65[x]	58.86±1.50[y]	55.78±0.95[y]
Val	81.18±1.56[a]	68.65±1.96[b]	78.26±0.85[a]	75.51±1.55[x]	67.70±1.13[y]	66.97±0.80[y]
Dispensable AA						
Ala	81.72±1.47[a]	65.41±2.20[c]	76.12±0.60[b]	76.86±1.33[x]	64.72±1.14[y]	68.17±0.72[y]
Asp	82.81±0.91	72.47±1.69	80.83±0.48	78.69±1.33	69.79±0.92	74.76±0.57
Cys	71.13±1.70[b]	64.25±2.15[c]	79.87±0.83[a]	57.07±2.00[y]	65.42±1.14[x]	66.52±0.74[x]
Glu	87.50±0.76	83.26±1.08	87.99±0.42	84.41±1.06	80.03±0.71	84.17±0.45
Gly	78.80±1.46	65.23±2.10	76.00±0.64	73.51±1.52	63.89±1.06	68.19±0.51
Pro	83.84±1.05	68.69±2.11	80.74±0.68	77.81±1.17	64.68±1.31	71.30±0.51
Ser	80.49±1.47[a]	66.52±2.42[b]	78.60±0.66[a]	75.53±1.46[x]	66.23±1.27[y]	69.51±0.63[x]
IAA	83.36±1.28	72.23±1.79	81.46±0.58	78.83±1.49	73.32±0.87	72.46±0.70
DAA	83.63±1.04[a]	74.81±1.61[b]	82.84±0.50[a]	79.17±1.27[x]	72.42±0.91[y]	76.61±0.51[xy]
Total AA	83.50±1.16[a]	73.53±1.70[b]	82.16±0.54[a]	79.00±1.38[x]	72.87±0.89[y]	74.56±0.60[y]

SBM = Soybean meal; CSM = Cottonseed meal; LCSM = Low-gossypol cottonseed meal; AA = Amino acids; IAA = Indispensable AA; DAA = Dispensable AA.

[a-c] and [x-z] represent the results of one-way ANOVA of those AA with significant interaction between diets and species. Means in a row with different superscripts within a species are significantly different ($P < 0.05$).

Data represent mean ± standard error of mean (SEM).

Means represent 6 replicates per species (broiler or hen) with 8 or 6 birds per replicate for broilers and hens, respectively.

Broilers were 21-day-old and layers were 35-wk-old.
