# Supplementary material for: Comparison of amino acid digestibility of soybean meal, cottonseed meal, and low-gossypol cottonseed meal between broilers and laying hens
Source: Anim Biosci. 2022 Sep 2;36(4):619–28. doi: 10.5713/ab.22.0073 (PMC9996273; doi:10.5713/ab.22.0073)
Supplement: Supplementary file 2 [file ab-22-0073-Supplementary-Table-2.pdf]

Table S2. The original data of standardized ileal digestibility of AA in broilers and laying hens fed different ingredient samples.

Item, %	Broiler			Laying hen		
	SBM	CSM	LCSM	SBM	CSM	LCSM
Indispensable AA						
Arg	89.49±0.88	87.33±0.86	91.19±0.39	88.54±1.20	86.56±0.61	87.88±0.55
His	86.43±1.15	77.69±1.47	83.87±0.57	83.65±1.39	76.92±1.00	77.50±0.81
Ile	85.43±1.10[a]	66.23±2.25[c]	79.73±0.58[b]	82.13±1.69[x]	71.07±1.27[y]	70.35±1.14[y]
Leu	85.59±1.10[a]	69.95±2.02[b]	81.99±0.53[a]	82.17±1.60[x]	73.73±1.15[y]	73.12±1.02[y]
Lys	85.82±1.42[a]	59.18±2.76[c]	73.53±0.63[b]	84.87±1.60[x]	69.05±1.22[y]	66.58±1.26[y]
Met	85.70±1.63[a]	65.16±2.40[b]	80.99±0.73[a]	81.41±2.26[x]	73.24±1.40[y]	71.66±1.12[y]
Met+Cys	80.62±1.63[a]	66.86±2.09[b]	82.58±0.76[a]	73.28±2.28	74.26±1.44	71.35±1.08
Phe	86.69±0.95	80.65±1.33	87.06±0.47	83.25±1.47	79.47±0.85	80.77±0.78
Trp	86.58±0.95[a]	72.45±1.86[b]	84.31±0.93[a]	81.90±1.68[x]	76.27±1.05[y]	73.90±1.04[y]
Thr	80.69±1.83[a]	61.18±2.98[b]	77.94±0.71[a]	76.69±1.74[x]	67.97±1.55[y]	65.78±1.16[y]
Val	83.82±1.56[a]	71.50±1.91[b]	81.44±0.82[a]	79.94±1.66[x]	72.50±1.27[y]	72.33±1.01[y]
Dispensable AA						
Ala	84.18±1.44	68.18±2.13	79.31±0.57	80.87±1.65	69.26±1.33	73.40±0.96
Asp	84.66±0.90	74.70±1.65	83.32±0.45	81.85±1.42	73.58±1.06	79.00±0.77
Cys	71.71±1.65[b]	68.03±2.15[c]	84.01±0.81[a]	65.50±2.50[y]	72.36±1.52[x]	74.13±1.35[x]
Glu	88.96±0.74	84.53±1.05	89.43±0.40	86.72±1.14	82.05±0.80	86.45±0.58
Gly	81.48±1.42	68.03±2.05	79.11±0.61	78.17±1.68	68.78±1.27	73.62±0.79
Pro	86.04±1.05	71.79±2.05	83.88±0.69	81.80±1.33	70.29±1.58	76.98±0.96
Ser	83.43±1.44[a]	69.91±2.38[b]	82.40±0.63[a]	80.90±1.60[x]	72.41±1.45[z]	76.44±0.93[y]
IAA	85.54±1.25[a]	74.42±1.74[b]	83.94±0.54[a]	82.50±1.59[x]	77.01±1.00[y]	76.63±0.88[y]
DAA	85.69±1.02	76.95±1.57	85.23±0.48	82.68±1.38	76.06±1.07	80.69±0.74
Total AA	85.62±1.13[a]	75.69±1.65[b]	84.59±0.51[a]	82.59±1.48[x]	76.53±1.04[y]	78.69±0.81[y]

SBM = Soybean meal; CSM = Cottonseed meal; LCSM = Low-gossypol cottonseed meal; AA = Amino acids; IAA = Indispensable AA; DAA = Dispensable AA.

[a-c] and [x-z] represent the results of one-way ANOVA of those AA with significant interaction between diets and species. Means in a row with different superscripts within a species are significantly different ($P < 0.05$).

Data represent mean ± standard error of mean (SEM).

Means represent 6 replicates per species (broiler or hen) with 8 or 6 birds per replicate for broilers and hens, respectively.

Broilers were 21-day-old and layers were 35-wk-old.
